# MicroRNAs as the pivotal regulators of Temozolomide resistance in glioblastoma

**DOI:** 10.1186/s13041-024-01113-6

**Published:** 2024-07-02

**Authors:** Mahsa Palizkaran Yazdi, Amirhosein Barjasteh, Meysam Moghbeli

**Affiliations:** 1 Department of Medical Genetics and Molecular Medicine, School of Medicine, Mashhad University of Medical Sciences, Mashhad, Iran; 2 Student Research Committee, Faculty of Medicine, Mashhad University of Medical Sciences, Mashhad, Iran

**Keywords:** Glioblastoma, Temozolomide, microRNA, Chemo resistance, Prognosis

## Abstract

Glioblastoma (GBM) is an aggressive nervous system tumor with a poor prognosis. Although, surgery, radiation therapy, and chemotherapy are the current standard protocol for GBM patients, there is still a poor prognosis in these patients. Temozolomide (TMZ) as a first-line therapeutic agent in GBM can easily cross from the blood-brain barrier to inhibit tumor cell proliferation. However, there is a high rate of TMZ resistance in GBM patients. Since, there are limited therapeutic choices for GBM patients who develop TMZ resistance; it is required to clarify the molecular mechanisms of chemo resistance to introduce the novel therapeutic targets. MicroRNAs (miRNAs) regulate chemo resistance through regulation of drug metabolism, absorption, DNA repair, apoptosis, and cell cycle. In the present review we discussed the role of miRNAs in TMZ response of GBM cells. It has been reported that miRNAs mainly induced TMZ sensitivity by regulation of signaling pathways and autophagy in GBM cells. Therefore, miRNAs can be used as the reliable diagnostic/prognostic markers in GBM patients. They can also be used as the therapeutic targets to improve the TMZ response in GBM cells.

## Background

Glioma is one of the aggressive nervous system tumors with a high rate of drug resistance [1, 2]. Glioblastoma (GBM) as the highest grade of astrocytoma is a malignant central nervous system disorder [3]. GBM is the primary cause of death among patients between the ages of 15 and 34 [4]. The current standard protocol for GBM therapy involves surgery, radiation therapy, and chemotherapy [5]. Apart from the therapeutic progresses in radiotherapy, chemotherapy, and surgical resection, there is still a poor prognosis in GBM patients [6, 7]. Resistance to therapeutic drugs is one of the most frequent causes of GBM recurrence [8, 9]. Temozolomide (TMZ) as a DNA-alkylating agent functions by promotion of DNA damage and double strand breaks (DSBs) that result in activation of caspase-mediated apoptosis in GBM cells [10–12]. Currently, regional fractionated radiation followed by TMZ are the first therapies for all GBM patients after surgery [13]. TMZ as a first-line therapeutic agent in GBM can easily cross from the blood-brain barrier to inhibit and induce the tumor cell proliferation and apoptosis, respectively [14]. However, due to the chemo and radiotherapeutic resistances, there is only a median survival of 14.6 months in GBM patients [15]. Regarding the limited repair mechanisms and anatomical complexity, treatment of the drug-resistant GBM is challenging [15–17]. Since, there are limited therapeutic choices for GBM patients who develop TMZ resistance; it is required to clarify the molecular mechanisms of chemo resistance to introduce the novel therapeutic targets. MicroRNAs (miRNAs) are involved in regulation of numerous biological processes such as autophagy, invasion, cell differentiation, proliferation, and apoptosis [18–20]. They function as either oncomiRs or tumor suppressive miRNAs during tumor progression [21, 22]. MiRNAs regulate chemo resistance through regulation of drug metabolism, absorption, DNA repair, apoptosis, and cell cycle [23, 24]. MiRNA deregulation has been found in GBM, which may be involved in tumor progression and therapeutic resistance [25–27]. Since, there are various reports about the miRNA profiles in TMZ-resistant GBM [28–32], we discussed the role of miRNAs in TMZ response through the regulation of signaling pathways, autophagy, and cell cycle to introduce them as the novel therapeutic options to improve prognosis among GBM patients (Table 1).

## Wnt/β-catenin and PI3K/AKT signaling pathways

**Fig. 1:**
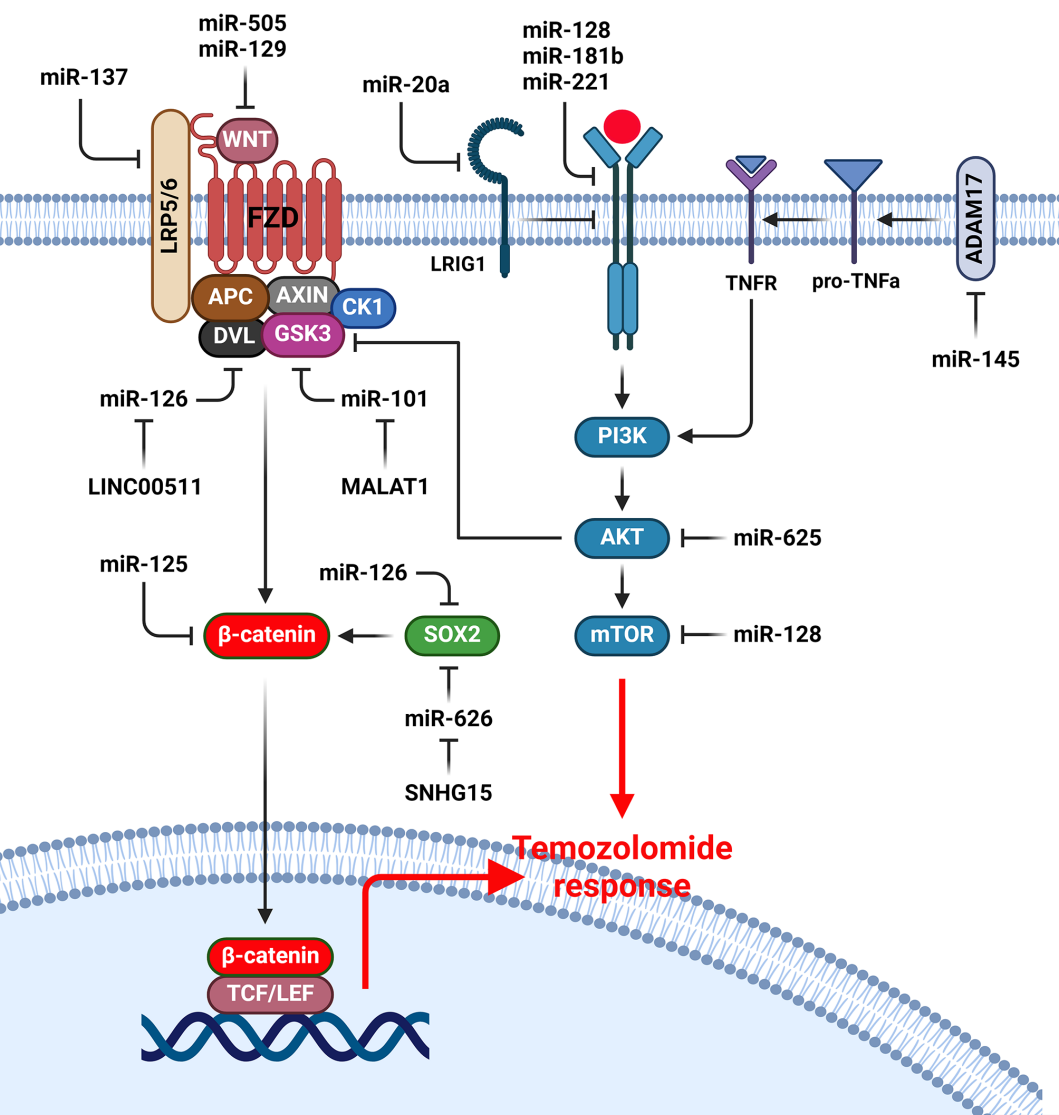
Role of miRNAs in TMZ response of GBM cells by regulation of Wnt/β-catenin and PI3K/AKT pathways. (Created with *BioRender.com*)

Wnt/β-catenin is an important oncogenic signaling pathway in tumor cells that can be triggered by the WNT ligands following the binding with FZD/LRP receptor [33]. WNT ligands promotes the accumulation of cytoplasmic β-catenin that finally enters to the nucleus to regulate WNT target genes [33]. It has been shown that miRNAs have a key role in TMZ response of GBM cells by regulation of Wnt/β-catenin pathway (Fig. 1). There was miR-505 down regulation in GBM tissues. It functioned as a tumor suppressor by targeting WNT7B. Moreover, TMZ had the ability to elevate the levels of miR-505 to enhance its inhibitory roles in GBM cells [34]. GSK3β as a component of degradation complex in WNT signaling regulates cell growth and survival [35–37]. MiR-101 increased TMZ sensitivity in GBM cells by GSK3β targeting. There was also a correlation between miR-101 down regulation and poor survival in GBM patients [38]. Wnt/β-catenin is a key regulator of EMT process in cancer [39–41]. There was miR-137 down regulation in recurrent GBM tissues. MiR-137 up regulated E-cadherin while down regulated vimentin and N-cadherin in GBM cells. MiR-137 increased TMZ sensitivity by targeting LRP6. Hypoxia-induced down regulation of miR-137 significantly regulated TMZ resistance and GBM growth via LRP6/β-catenin pathway [42]. LINC00511 activated Wnt/β-catenin via miR-126-5p sponging. There was a correlation between LINC00511 up regulation and poor prognosis in GBM patients. LINC00511/miR-126-5p/DVL3 axis induced the TMZ resistance in GBM cells [43]. The downstream pathways of Wnt5a are planar cell polarity (PCP) and Wnt/Ca2 + pathways, which have a key role in cell physiology and tumor progression [44, 45]. PCP pathway activates JNK through the regulation of morphogenetic motions and cell polarity. Wnt/Ca2 + pathway activate PKC and cam kinase II, which can modulate cell adhesion and motility [44–46]. PKC/ERK/NF-κB and JNK pathways were activated by dysregulation of miR-129-5p/Wnt5a signaling, resulting in a more malignant phenotype with TMZ resistance. MiR-129-5p suppressed angiogenesis, tumor invasion, and TMZ resistance by Wnt5a targeting in GBM cells [47]. SOX2 up regulation is observed in various cancers that increases tumor aggressiveness and poor prognosis [48]. It regulates glioma cell invasion and proliferation as an oncogene [49, 50]. In addition, SOX2 has been linked with the development of resistance to several chemotherapeutic medications by regulating numerous signaling pathways [51–53]. SOX2 is involved in regulation of cisplatin response in tumor cells by regulation of Wnt-β-catenin pathway [54]. The decreased expression of miR-126-3p was observed in GBM cells and samples that exhibited TMZ resistance. MiR-126-3p enhanced the GBM sensitivity to TMZ by SOX2 targeting and inhibition of the Wnt/β-catenin pathway [55]. SOX2 mediated miR-486-5p expression enhanced the self-renewal capacity of GBM by PTEN and FoxO1 down regulations [56]. Glioma-associated microglial cells (GAMs) interact closely with GBM cells through intracellular communication and share similar functions with tumor-associated macrophages in the peripheral system [57, 58]. GAMs release various signaling molecules and cytokines that inhibit apoptosis, while promote metastasis and angiogenesis [59, 60]. TMZ-resistant GBM samples had a notable SNHG15 up regulation, which was correlated with the aggressive characteristics of GBM. Elevated expression of lncSNHG15 was associated with increased levels of stemness markers such as β-catenin and Sox2, as well as oncogene markers such as EGFR and CDK6. Glioma-associated microglia (M2-GAMs) could be more easily M2-polarized by TMZ-resistant GBM cells than by their sensitive counterparts. SNHG15 down-regulation led to a decrease in carcinogenesis, self-renewal, and heightened TMZ sensitivity. TMZ resistance could be overcome through SNHG15/CDK6/miR-627 by reducing the M2 polarization of glioma-associated microglia in GBM [61].

Growth factors activate the PI3K/AKT pathway through binding with receptor tyrosine kinases (RTKs) that results in cell growth, proliferation, drug resistance, and tumor progression [62, 63]. It has been reported that miRNAs have an important role in TMZ response of GBM cells via regulation of PI3K/AKT pathway (Fig. 1). RTKs promote resistance to both chemotherapy and irradiation in GBM cells [64–66]. EGFR belongs to the HER family of RTKs [67, 68]. Growth factors bind with EGFR to activate downstream pathways that regulate differentiation, cell growth, and survival [69, 70]. MiR-181b increased TMZ sensitivity in GBM cell by regulation of the EGFR pathway [71]. There was EGFR down regulation in TMZ and irradiation-resistant GBM cells. MiR-221 increased TMZ and radio therapeutic resistances in GBM cells by EGFR targeting [72]. CircCABIN1 induced TMZ resistance and stemness in GBM cells by miR-637 sponging and subsequent OLFML3 up regulation that activated ErbB pathway [73]. LRIG1 as an inhibitor of EGFR promoted the TMZ sensitivity in GBM cells [74]. MiR-20a mediated TMZ resistance by suppressing LRIG1 in GBM cells [75]. c-Met as a RTK is involved in embryogenesis [76]. Deregulation of c-Met promoted cell proliferation and angiogenesis, while inhibited apoptosis in brain tumors [77]. Overexpression of c-Met affects chemo sensitivity, causing GBM cells to become resistant to drugs [78, 79]. Moreover, miR-128-3p prevents the EMT process through vimentin down regulation while CDH1 up regulation. MiR-128-3p promoted the inhibitory role of TMZ in cell migration and proliferation via EMT suppression. miR-128-3p induced the TMZ sensitivity of GBM cells through c-Met targeting and EMT suppression [80]. AKT2 as a serine/threonine kinase has key roles in tumor metastasis, metabolism, radio-resistance, and drug resistance [81]. Down regulation of miR-625 was observed in human glioma in contrast to normal human brain tissues. MiR-625 increased apoptosis while reduced the TMZ resistance of glioma cells by targeting AKT2 [82]. Activation of Akt can efficiently suppress GSK-3β, leading to a decrease in β-catenin degradation [83]. Down regulation of β-catenin also suppresses cell growth while induces cell death. CD133(+) glioma cells have demonstrated a significant self-renewal ability, resulting in subcutaneous tumors and the generation of stem cell spheres in nude mice [84]. The concurrent administration of LY294002 (PI3K inhibitor), TMZ, and miR-125b inhibitor demonstrated the most significant impact on p-β-catenin up regulation while reduction of p-GSK-3β. This suggests that the combined treatment was highly influential in inactivation of Wnt/β-catenin pathway. TMZ resistance of Glioblastoma stemcells (GSCs) cells could be successfully reversed by treating them with both miR-125b inhibitors and PI3K/Akt inhibition [85]. MALAT1 inhibition increased TMZ sensitivity in GBM cells by miR-101/GSK3β and MGMT [86]. IGF signaling among the several other tumor microenvironmental factors has been found to increase the risk of brain tumor progression [87]. The binding of the IGF-1 with IGF-1R results in its activation by autophosphorylation that promotes cell migration and proliferation in gliomas [88]. IGF-1 also reduced etoposide-induced apoptosis of glioma cells via Bcl-2 up regulation while inhibition of caspase-3 activity [89]. Mammalian target of rapamycin (mTOR) belongs to the serine/threonine kinases that regulate cell growth and proliferation [90]. IGF-1 trigger the mTOR and its downstream targets such as 4E-BP1 and p70S6K1 via the PI3K/PDK1/AKT axis [91]. mTORC1 regulates protein translation in the brain to control learning, memory, synaptic plasticity, and the pathogenesis of GBM [92]. The production of ROS, MMP loss, apoptosis, and non-protective autophagy were significantly increased by miR-128 via mTOR, PIK3R1, IGF1, and RICTOR targeting. Temozolomide can promote apoptosis in glioblastoma cells by miR-128 up regulation. TMZ increased apoptosis through JNK2/c-Jun mediated miR128 up regulation in GBM cells. MiR-128 targeted p70S6K1 and down regulated its substrates such as HIF-1 and VEGF [93]. HOXA-AS2 promoted TMZ resistance through regulation of miR-302a-3p/IGF1 axis in GBM cells [94]. Disintegrin and metalloproteinase-17 (ADAM17) has ability to cleave the membrane-bound TNF-α which activates EGFR pathway [95, 96]. However, the function of ADAM17 goes beyond the release of soluble TNF-α and is able to process various substrates, such as EGFR, cytokines, and adhesion molecules [97, 98]. Furthermore, ADAM17 is responsible for pathological and physiological processes, such as cell growth, inflammation, differentiation, regeneration, and tumor progression [97]. It was identified that miR-145-5p increased TMZ sensitivity in GBM cells by ADAM17 targeting [99].

### TGF-β, NF-kB, and hedgehog signaling pathways

**Fig. 2:**
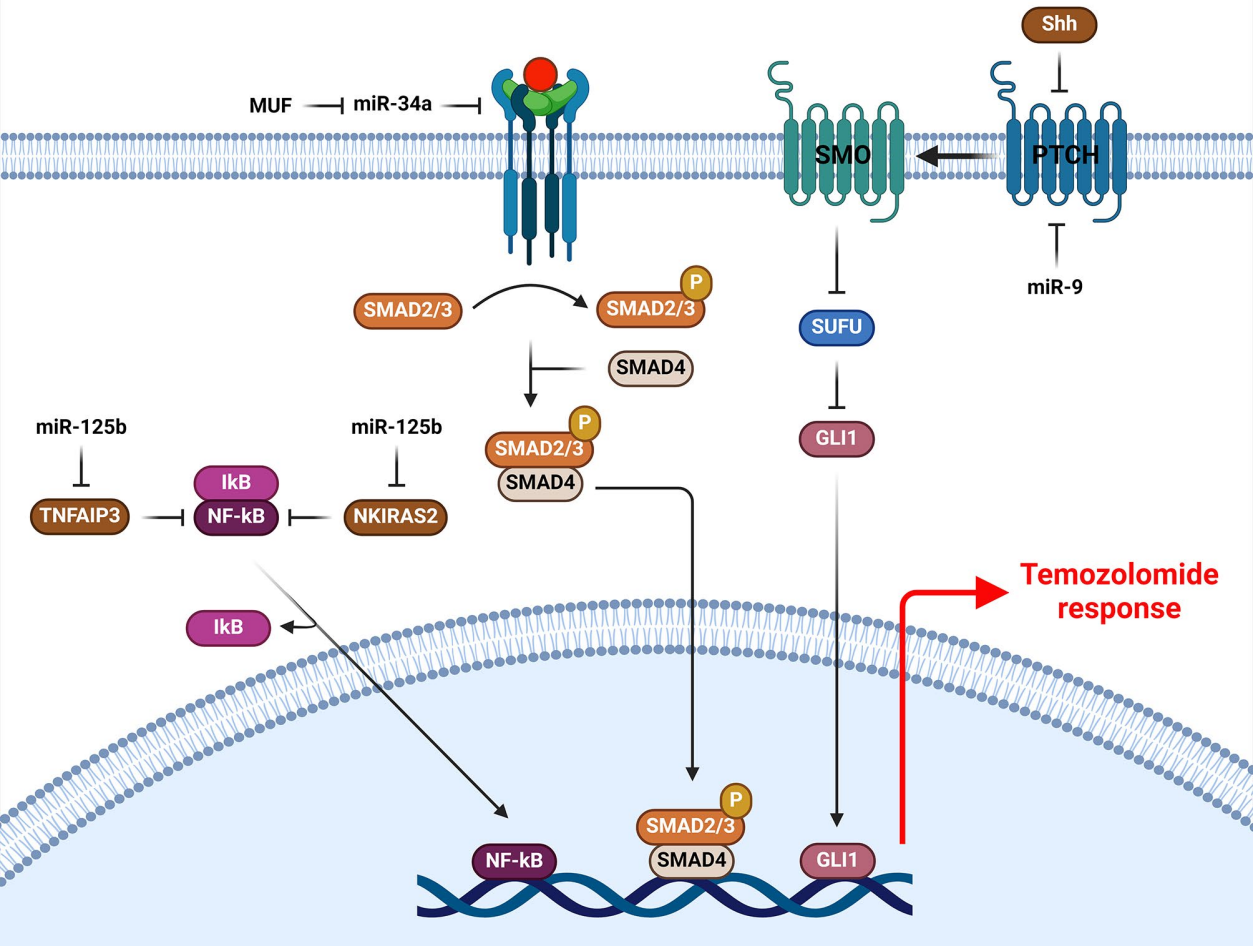
Role of miRNAs in TMZ response of GBM cells by regulation of TGF-β, NF-kB, and hedgehog pathways. (Created with *BioRender.com*)

MiRNAs have a key role in TMZ response of GBM cells by regulation of TGF-β, NF-kB, and Hedgehog signaling pathways (Fig. 2). TGF-β is a multifaceted regulatory cytokine with cell proliferation, differentiation, and tissue homeostasis functions [100, 101]. It enhances the GBM growth, metastasis, and angiogenesis. TGF-β enables GBM cells to evade growth suppression and immune checkpoint blockade and develop resistance to chemotherapy [102–104]. There was lncRNA-MUF up regulation in GBM tissues that was correlated with histological grading. It serves as an oncogenic lncRNA and sponges miR-34a which suppresses Snail1 to promote glioma cell growth and invasion. Inhibition of lncRNA-MUF increased TMZ-mediated apoptosis in GBM cells by reducing TGF-β-induced phosphorylation of SMAD2/3 [105]. The most direct cause of drug tolerance is the expression of Methylguanine DNA methyltransferase (MGMT), as it reverses DNA alkylation in TMZ-induced O6-methyguanine lesions by eliminating methyl groups [106, 107]. Patients with higher levels of MGMT had less effective outcomes from TMZ chemotherapy in comparison to those with lower MGMT levels [108]. It was shown that TGF-β1 was correlated with TMZ resistance in GBM cells with MGMT hypomethylation. TGF-β1 up regulated the lncRNAs that attached competitively to KSRP, thereby blocking KSRP from taking part in switching of miR-198 and finally up regulated MGMT. H19 or HOXD-AS2 sponged KSRP and prohibited it from engaging in the FSTL1/miR-198 cascade, therefore resulting in miR-198 down regulation and MGMT up regulation. Consequently, HOXD-AS2 or H19 are responsible for TMZ resistance through KSRP/miR-198/MGMT axis. TGF-β1 up regulated the MGMT by miR-198 inhibition that conferred TMZ resistance in GBM cells [104]. MGMT can be regulated by promoter methylation, transcription factors, histone acetylation, and microRNAs [109]. There was miR-198 down regulation in GBM tissues that was correlated with poor prognosis. MiR-198 enhanced the TMZ sensitivity in GBM cells via MGMT targeting [110]. NF-kB has a key role in chemo resistance of malignant tumor cells [111, 112]. The inactivated NF-kB comprises p50 (NFKB1)/p65 (RelA) subunits. Regularly, they are held in the cytoplasm by IkB, which is the NF-kB inhibitor. External stimuli or stress activate the IKK to phosphorylate IkB for protein degradation through ubiquitin. Subsequently, the activated NF-kB migrates to the nucleus and interacts with different genes which affect apoptosis, invasion, and proliferation. Tumor necrosis factor alpha-induced protein 3 (TNFAIP3) negatively operates in a feedback loop to prevent NF-kB from being activated. This protein also catalyzes the fragmentation of ubiquitin chains linked to K63 and the attachment of K48-linked polyubiquitin chains, which aids in the degradation of receptor-interacting serine-threonine kinase 1 [113, 114]. NF-kB inhibitors interacting with RAS-like (NKIRAS) 1 and 2 intervene with IkB proteasomal degradation and are implied in the activity of NF-kB as well [115–118]. MiR-125b induced TMZ resistance by TNFAIP3 and NKIRAS2 targeting in GBM cells [119]. The activation of SMO is triggered by the interaction between SHH and PTCH1 receptor, which results in the de-repression of SMO [120]. Glioma-associated oncogene 1 (Gli1), which is a transcriptional factor and downstream of SMO, is the main player in the SHH signaling pathway. Several cancers have been associated with the overexpression of SHH signaling [121]. MiR-9 induced TMZ resistance by PTCH1 targeting that up regulated the drug efflux pumps in GBM cells [122].

### MAPK signaling pathway

**Fig. 3:**
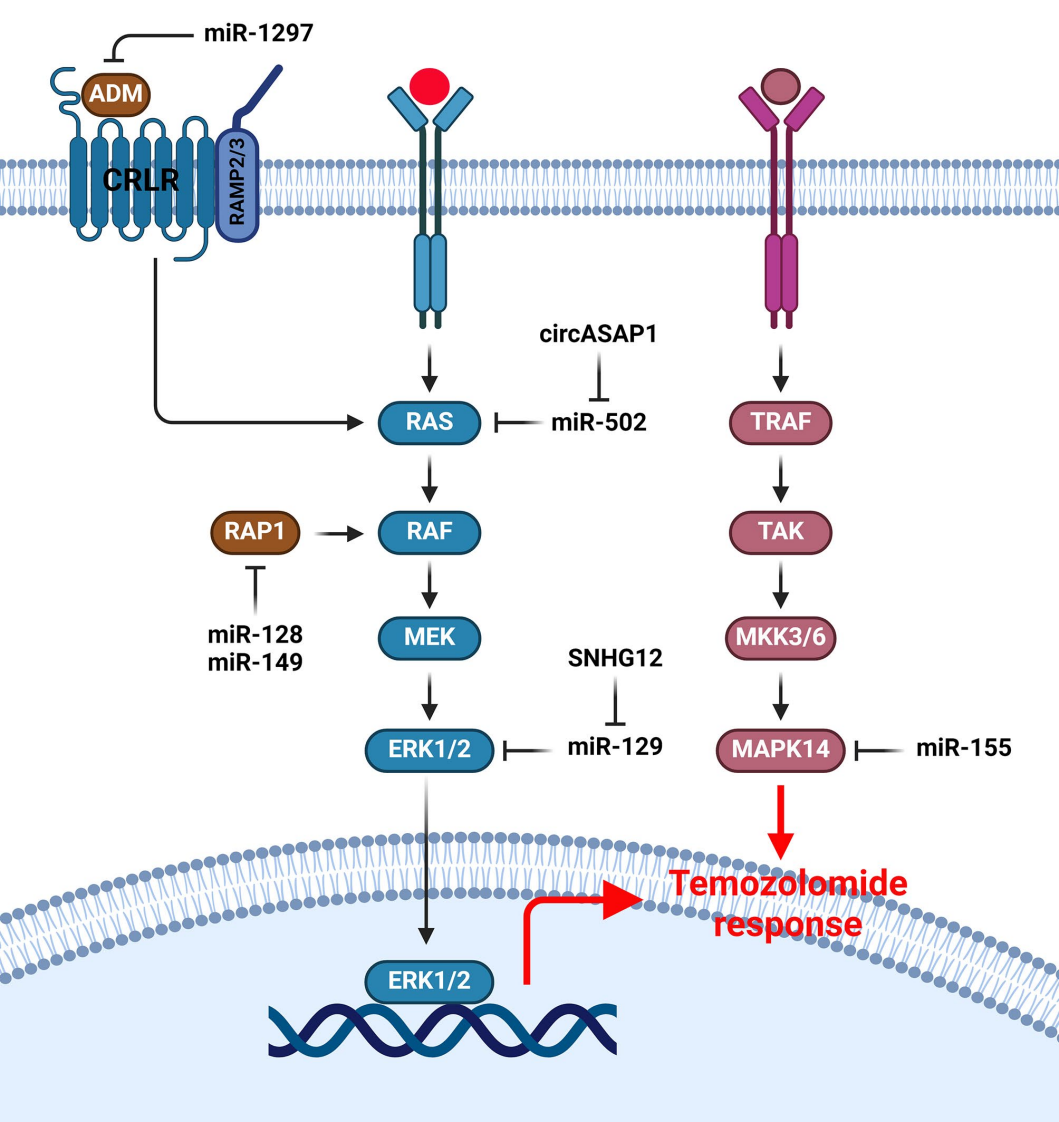
Role of miRNAs in TMZ response of GBM cells by regulation of MAPK pathway. (Created with *BioRender.com*)

MAPK/ERK is a key pathway during tumor progression that can be activated by the external mitogens and growth factors [123]. It has been reported that miRNAs have key roles in TMZ response of GBM cells by regulation of MAPK pathway (Fig. 3). MAPK1 is involved in chemo resistance and malignant phenotype in various cancers [124, 125] .E2F7 induces glioma cell proliferation [126, 127]. It was shown that SNHG12 was epigenetically activated by DNA methylation that regulated the MAPK/ERK pathway and cell proliferation by miR-129-5p sponging in GBM cells. SNHG12 increased TMZ resistance by miR-129-5p sponging and subsequent MAPK1 and E2F7 up regulations [128]. NRAS is an important member of the RAS family, which functions as an on/off switch through GDP/GTP regulation. It is a membrane-bound protein that has a vital function in the signal transduction mechanisms of hormones, cytokines, and growth factors. NRAS regulates tumor cell proliferation and is often overstimulated in various cancers [125, 129]. CircASAP1 induced TMZ resistance by miR-502-5p sponging and NRAS up regulation that activated MEK1/ERK1-2 signaling. EIF4A3-mediated circASAP1 increased TMZ resistance and tumor progression in GBM cells [130]. RAP1 is a Ras GTPase that regulates cellular adhesion, growth, and migration. Two highly related isoforms of RAP1 are RAP1B and RAP1A [131]. Rap1B is linked to the cytoskeleton during cell activation [132]. Rap1B down regulated is correlated with reduced glioma cell migration that is induced by lysophosphatidic acid [133]. Cell division cycle 42 (Cdc42), RhoA, and Rac1 are also Rho family members that have key roles cell migration, adhesion, and actin cytoskeletal reorganization [134, 135]. MiR-128 and miR-149 improved TMZ sensitivity in GBM cells through Rap1B-mediated cytoskeletal remodeling. There was miR-149 down regulation in GBM tissues that was correlated with grades of astrocytomas. MiR-128 and miR-149 reduced cell invasion and proliferation by Rap1B regulation in GBM cells [136]. Adrenomedullin (ADM) as a vasodilator hormone significantly affects many vital pathways including PI3K/Akt and ERK. ADM triggers relaxation of blood vessels through PI3K/Akt signaling that is mediated by the endothelium [137], and its infusion alleviates reperfusion injury or myocardial ischemia [138]. ADM plays a regulatory role in modulating various downstream pathways that promote the proliferation and viability of endothelial cells through MAPK/ERK activation [139]. Moreover, ADM has the ability to increase the expression of Bcl-2 by autocrine or paracrine mechanisms of action to protect cancer cells from hypoxia-mediated apoptosis [140]. An up regulation of ADM was observed in TMZ resistant glioma tissues and cells. ADM inhibition promoted TMZ effects on Bax/Bcl-2, ERK1/2, and Akt phosphorylation. Moreover, miR-1297 induced TMZ sensitivity in glioma cells by ADM targeting [141]. MAPK14 could decrease the accumulation of reactive oxygen species, which can subsequently prevent hepatocarcinogenesis and liver fibrogenesis [142]. The p38-MAPK pathway has essential role in cell response to stress and cancer [143]. MiR-155 knockdown reduced cell invasion by p38 targeting. MiR-155 induced MMP9 and MMP2 secretions in the SF767 cell supernatant. Moreover, miR-155 knockdown improved the anti-tumor effect of TMZ on gliomas via MAPK14 and MAPK13-induced ROS generation [144].

### Autophagy and cell cycle

Autophagy is a conserved cellular process that is responsible for degradation of intracellular proteins and organelles [145, 146]. It can be activated in both normal and stress condition to provide required metabolic substrates for the cell survival. Autophagy is also activated to preserve cellular homeostasis in infection, aging, neurodegenerative diseases, myopathies, and cancer [147, 148]. The mechanism of autophagy is the creation of autophagosome which merges with lysosomes to produce autolysosomes for intracellular degradation [149, 150]. Autophagy is activated following the TMZ treatment that results in chemo resistance in tumor cells [151–153]. In certain instances, autophagy induces TMZ-mediated apoptosis in GBM cells. Additionally, rapamycin, which induces autophagy, could increase apoptosis caused by chemotherapy [154–158]. STAT3 as a transcription factor regulates the autophagy from autophagosome formation to maturation [159]. MiR-519a down regulation was observed in TMZ resistant GBM tissues and cells. It improved the TMZ response of GBM cells through enhanced GBM cell autophagy by facilitating the separation of the Bcl-2/Beclin-1 complex. Moreover, miR-519a induced autophagy by suppressing the STAT3/Bcl-2 axis [160]. MiR-17 regulated autophagosome formation via ATG7 targeting that improved TMZ sensitivity in GBM cells [161]. MiR-30a significantly reduced TMZ-mediated autophagy while induced apoptosis by BECN1 targeting in GBM cells [162].

Hypoxia has been suggested to cause resistance to chemotherapy or radiotherapy in a number of malignant tumors [163, 164]. Critical cellular responses to hypoxia include the stability and activation of HIF1α and HIF2α that have a vital role in tumor progression [165, 166]. It is speculated that hypoxia supports the preservation of GSCs’ undifferentiated status and resistance to treatment as they often reside in hypoxic microenvironments [167]. GSCs interact with immune cells, astrocytes, vascular cells, and neurons in the hypoxic microenvironment to support the tumor maintenance. RHOB belongs to the Rho small GTPase family that has a vital role in regulation of apoptosis and cell cycle progression [168, 169]. Hypoxia may trigger RHOB via GSK-3 in GBM cells [170]. MiR-30b-3p targeted RHOB, which decreased cell cycle arrest by CDK6 and CDK2 up regulations while reduced apoptosis by BCL-2 up regulation and Bax down regulation. HIF-1α and STAT3 transcriptionally enhanced the expression of miR-30b-3p in GSCs under hypoxic conditions. MiR-30b-3p increased TMZ resistance by RHOB targeting. miR-30b-3p up regulation was correlated with poor response to TMZ in GBM tissues [171]. WEE1 kinase as a G2/M checkpoint arrest has key role for pre-mitotic DNA repair [172]. FOXD3-AS1 conferred TMZ resistance through miR-128-3p/WEE1 axis in GBM cells [173]. MiR-125b induced TMZ resistance by STAT3 targeting in GSC cells [174].

## Conclusions

TMZ is the first-line therapeutic agent in GBM; however there is a high rate of TMZ resistance among GBM patients. Since, there are limited therapeutic choices for GBM patients who develop TMZ resistance; it is required to clarify the molecular mechanisms of chemo resistance to introduce the novel therapeutic targets. In the present review we discussed the role of miRNAs in TMZ response of GBM cells. It has been reported that miRNAs mainly increased TMZ sensitivity by regulation of signaling pathways and autophagy in GBM cells. Therefore, miRNAs can be used as the reliable tumor markers and therapeutic targets in GBM patients. Regarding the role of miRNAs as the TMZ sensitizers, a miRNA mimic strategy can be suggested to increase the TMZ response among GBM patients. However, further clinical trials and animal studies are needed to use the miRNAs as the therapeutic targets to improve the TMZ response in GBM patients.

**Table 1:** Role of miRNAs in TMZ response of GBM cells

miRNA	Target	Samples	TMZ response	Clinical Application	Study
miR-221	DNM3	SHG-44, U87MG, HEB, and U251 cell lines	Increased TMZ resistance	Diagnosis	Yang [29]
miR-129	IGF2BP2	87 patients U251, U87, A172, and SHG-44 cell lines	Increased TMZ sensitivity	Diagnosis	Wang [32]
miR-505	WNT7B	41 patients U251, U87, LN229, U373, A172, and NHA cell lines	Increased TMZ sensitivity	Diagnosis	Zhang [34]
miR-137	LRP6	59 patients U251 and U87 cell lines	Increased TMZ sensitivity	Diagnosis	Li [42]
miR-129	WNT5A	16 patients U251, LN229, A172, LN18, and T98G cell lines	Increased TMZ sensitivity	Diagnosis & prognosis	Zeng [47]
miR-126	SOX2	80 patients U87 and U251 cell lines	Increased TMZ sensitivity	Diagnosis & prognosis	Luo [55]
miR-181b	EGFR	20 patients U87 and U251 cell lines	Increased TMZ sensitivity	Diagnosis	Chen [71]
miR-20a	LRIG1	U251 cell line	Increased TMZ resistance	Diagnosis	Wei [75]
miR-128	C-MET	24 patients U87, T98, LN229, U251, and A172 cell lines	Increased TMZ sensitivity	Diagnosis	Zhao [80]
miR-625	AKT2	26 patients U87, LN229, U251, A172, and U118 cell lines	Increased TMZ sensitivity	Diagnosis	Zhang [82]
miR-101	GSK3B	U251 cell line	Increased TMZ sensitivity	Diagnosis	Cai [86]
miR-302a	IGF1	264 patients U87 and U251 cell lines	Increased TMZ sensitivity	Diagnosis	Lin [94]
miR-145	ADAM17	DBTRG-05MG, M059K, and U87MG cell lines	Increased TMZ sensitivity	Diagnosis	Yang [99]
miR-198	MGMT	30 patients A172, U87, U251, U118, LN229, U138, and T98 cell lines	Increased TMZ sensitivity	Diagnosis & prognosis	Nie [110]
miR-9	PTCH1	U87, T98G, BT145, and BT164 cell lines	Increased TMZ resistance	Diagnosis	Munoz [122]
miR-502	NRAS	50 patients N3S and U251 cell lines	Increased TMZ sensitivity	Diagnosis	Wei [130]
miR-128 mir-149	RAP1B	U251 and U87 cell lines	Increased TMZ sensitivity	Diagnosis & prognosis	She [136]
miR-1297	ADM	18 patients U87 and T98G cell lines	Increased TMZ sensitivity	Diagnosis & prognosis	He [141]
miR-155	P38	U251, U87, A172, SF767, SF126, and SHG-44 cell lines	Increased TMZ resistance	Diagnosis	Liu [144]
miR-519a	STAT3 BCL2	48 patients U87 cell line	Increased TMZ sensitivity	Diagnosis	Li [160]
miR-17	ATG7	T98G and U373MG cell lines	Increased TMZ sensitivity	Diagnosis	Comincini [161]
miR-30a	BECN1	U251 cell line	Increased TMZ sensitivity	Diagnosis	Xu [162]
miR-30b	RHOB	60 patients	Increased TMZ resistance	Diagnosis	Yin [171]
miR-128	WEE1	A172, U87, U251, LN118, and T98 cell lines	Increased TMZ sensitivity	Diagnosis	Ling [173]

## Data Availability

The datasets used and/or analyzed during the current study are available from the corresponding author on reasonable request.

## Abbreviations

ADM: Adrenomedullin
Cdc4: Cell division cycle 42
ADAM1: Disintegrin and metalloproteinase-17
DSBs: Double strand breaks
GBM: Glioblastoma
GSCs: Glioblastoma stemcells
GAMs: Glioma-associated microglial cells
Gli1: Glioma-associated oncogene 1
mTOR: Mammalian target of rapamycin
MGMT: Methylguanine DNA methyltransferase
miRNAs: MicroRNAs
NKIRAS: NF-kB inhibitors interacting with RAS-like
PCP: Planar cell polarity
RTKs: Receptor tyrosine kinases
TMZ: Temozolomide
TNFAIP: Tumor necrosis factor alpha-induced protein 3
